# Genotype-specific retinal and choroidal perfusion patterns in inherited retinal diseases: an SS-OCTA analysis

**DOI:** 10.1186/s40942-025-00706-0

**Published:** 2025-07-23

**Authors:** Yu Rong, Junfeng Li, Jianquan He, Daowei Zhang, Jiawen Wu, Hongli Liu, Ting Li, Ping Xu, Qing Chang, Jihong Wu

**Affiliations:** 1 Department of Ophthalmology, Eye and ENT Hospital, Fudan University, Shanghai 200031, China; 2 Shanghai Key Laboratory of Visual Impairment and Restoration,, Science and Technology Commission of Shanghai Municipality, Shanghai 200031, China; 3 Key laboratory of Myopia and Related Eye Diseases, NHC, Shanghai 200031, China; 4 Key Laboratory of Myopia and Related Eye Diseases, Chinese Academy of Medical Sciences, Shanghai 200031, China

**Keywords:** Inherited retinal diseases, Retinitis pigmentosa, SS-OCTA, Genotype, Perfusion density, Foveal avascular zone, Choroidal vascularity index

## Abstract

**Background:**

Retinitis pigmentosa (RP), an inherited retinal disease, is characterized by progressive vision loss driven by the gradual degeneration of retinal photoreceptors. This process manifests as impaired dark adaptation, night blindness, constriction of the visual field, and the deterioration of central vision. Although the progression can be monitored by electroretinography (ERG), visual field (VF) tests and optical coherence tomography (OCT) to some extent, it’s hard to achieve high repeatability. Considering the correlation between patients’ retinal blood volume and their visual function, OCT angiography (OCTA) can be a good choice for monitoring RP progression by objectively quantifying vascular changes.

**Methods:**

This study included 62 patients and 21 matched controls. Patients with RP were classified into five groups based on their genotype (*CYP4V2*, *EYS*, *PRPH2*, *RPGR*, and *USH2A*). Quantitative measurements and analyses were performed in nine fields of the fundus.

**Results:**

Defects were observed in each layer among all RP groups, showing different patterns of damage to the vasculature of the SCP, DCP, CC, and MLC. Foveal avascular zone (FAZ) sizes of the SCP and DCP in *CYP4V2* and *EYS* groups, respectively, were larger than those in healthy individuals; PDs were associated with retinal function in each group. The CVI decreased to various degrees based on genotype and was associated with retinal function.

**Conclusion:**

Patients with RP had decreased PDs in the retina and choroid. PDs correlated with specific genotypes and retinal functions. SS-OCTA may be a non-invasive method for detecting the severity of RP.

**Supplementary Information:**

The online version contains supplementary material available at 10.1186/s40942-025-00706-0.

## Background

Retinitis pigmentosa (RP) is an inherited retinal disease characterized by progressive vision loss caused by the gradual breakdown of photoreceptors in the retina, which leads to impaired dark adaptation, night blindness, visual field constriction, and the deterioration of central vision [1]. Although RP is generally diagnosed during childhood or early adulthood, it can also affect adults. Progression can be monitored through electroretinography (ERG) and visual field (VF) tests [2]. Unfortunately, both tests have failed to achieve high repeatability [3]. Structural optical coherence tomography (OCT) has been used to monitor the progression of RP by measuring the ellipsoidal zone area and shows better repeatability than ERG and VF. Moreover, imaging with OCT angiography (OCTA) may optimize the ability to monitor the progression of RP by objectively quantifying vascular changes, since retinal blood volume in patients with RP correlates with visual function. Further research into the retinal and choroidal vessels of patients with RP may promote a better understanding of the underlying pathology of the condition, as changes in the retinal vasculature with vessel attenuation observed on fundoscopy are hallmarks of the disease [4]. 

Previous studies have evaluated ocular hemodynamics and identified a reduction in ocular blood flow in patients with RP. By studying the retinal vasculature, researchers can determine the extent of damage to the retinal and choroidal layers and how that damage may contribute to the progression of RP [5]. Additionally, studies on retinal and choroidal vessels can provide insights into the mechanisms of RP and help guide the development of treatments for this condition; however, the relationship between ocular hemodynamics and RP is not fully understood. Widefield OCTA technology is frequently used to explore vascular disorders of the retina from the macular region to the periphery through fundus imaging [6, 7]. OCTA is expected, therefore, to help researchers obtain a more comprehensive picture of the vascular changes that occur in patients with RP. This study used SS-OCTA to evaluate the PD of SCP, DCP, CC), and MLC, as well as the CVI in the central, mid-peripheral, and peripheral fields of patients with RP carrying mutations in the *CYP4V2*,* EYS*,* PRPH2*,* RPGR*, or *USH2A* genes.

## Materials and methods

### Study participants

In this prospective observational study, 62 patients previously diagnosed with varying stages of RP and 21 healthy individuals were referred to the Eye and ENT Hospital of Fudan University in Shanghai, China, between March and September 2022. The protocol for this study was approved by the Eye and ENT Hospital of Fudan University and adhered to the principles of the Declaration of Helsinki.

We performed whole-exome sequencing (WES) or whole-genome sequencing (WGS) to identify RP-associated homo- or heterozygous mutations in the *CYP4V2*, *EYS*, *PRPH2*, *RPGR*, and *USH2A* genes of patients with RP. Genomic DNA was extracted by the MagPure Buffy Coat DNA Midi KF kit according to the instructions. High-throughput sequencing was performed using the DNBSEQ-T17 in PE100 + 10 mode to obtain the raw sequencing data. Raw sequencing reads were evaluated for quality by LUSH_Aligner. SNV (single-nucleotide variant) and Indel (insertion/deletion) calling were performed using LUSH_BQSR and LUSH_HC. Detected variants were annotated and filtered against multiple databases: NCBI dbSNP v147, dbNSFP v2.9.1, ESP6500 v2 HapMap, 1000 Genomes Project, and an in-house database of 100 healthy Chinese adults. Suspected pathogenic variants were prioritized using BGI’s proprietary algorithms for functional prediction and frequency filtering. Variants were interpreted using disease databases (ClinVar, OMIM, HGMD) and classified according to ACMG guidelines [8, 9]. 

Patients were diagnosed with RP according to the following criteria: typical symptoms of night blindness or visual acuity loss; typical fundus appearance; visual field loss; reduced or absent function of photoreceptors measured with an electroretinogram [10, 11]. 

The exclusion criteria for the RP and control group were as follows: patients with a complex or unclear genetic diagnosis; fewer than five patients with the same disease-causing gene; diagnosis of other ocular disorders; the presence of disease complications such as nystagmus, severe cataract(s), retinal detachment, or edema; patients with high myopia (SE < -6.00D); previous ocular trauma or surgery; and the inability to undergo a comprehensive ophthalmologic examination. Clinical, genetic, and electrophysiological diagnoses of RP were made for all patients. The participants of the control group were healthy participants without a clinical or genetic diagnosis of retinitis pigmentosa, at the same time, matched for age and sex with the RP group.

All patients with RP underwent a comprehensive ophthalmologic examination using standard protocols, including measurement of the best-corrected visual acuity (BCVA), visual field (VF) test (Humphrey Field Analyzer; Carl Zeiss, Dublin, CA, USA) with a reliable VF defined as fixation loss (FL) and false-positive (FP) rates < 20% and < 15%, respectively, intraocular pressure (IOP) assessment, fundus examination, color fundus photography, autofluorescence, SS-OCTA (BM-400 K BMizar; TowardPi Medical Technology Co., Ltd., Beijing, China), and OCT (Heidelberg Spectralis OCT, Heidelberg Engineering, Heidelberg, Germany). The patients were divided into the following five groups based on the pathogenic genes identified through WES or WGS: *CYP4V2*, *EYS*, *PRPH2*, *RPGR*, and *USH2A*.

### OCTA image acquisition and analysis

Two well-trained ophthalmologists acquired and analyzed patient images using the newly developed SS-OCTA equipment. Before imaging, each patient underwent pupillary mydriasis. For each eye, a 24 mm × 20 mm OCTA scan was acquired. Low-quality images (score < 7) with significant motion artifacts or extensive incorrect segmentation were excluded. A higher-order moment amplitude-decorrelation angiography algorithm was applied to detect motion signals. This technique visualizes the vasculature involved in retinal circulation by capturing higher-order statistical signals from the OCTA data. Each layer was recognised using artificial intelligence. The PD of the SCP, DCP, CC, and MLC was evaluated, and the layers were differentiated through manual and automatic segmenting correction of the SS-OCTA images using the software developed for the equipment. The inner plexiform layer (IPL) and outer plexiform layer (OPL) captured on the OCT images were utilised as anatomical references during manual adjustments for the stratification of the superficial and deep retinal vessel layers. The CC, the sinusoidal capillary network of the choroidal circulation adjacent to Bruch’s membrane, was differentiated from the MLC using structural references.

The entire fundus was used to calculate the mean PD of each layer. The layers were then equally divided into the following nine parts, with the PD acquired for each: temporal superior (part1), superior (part2), nasal superior (part3), temporal (part4), central (part5), nasal (part6), temporal inferior (part7), inferior (part8), and nasal inferior (part9). Area and circumference measurements were used to quantify the size of the FAZ.

### Statistics

The Shapiro-Wilk test was used to evaluate the departure of continuous variables from the normal distribution, and differences in continuous data were tested using the Mann-Whitney U or Bonferroni test. Categorical variables are expressed as frequencies and percentages, while continuous variables are expressed as means and standard deviations or medians and interquartile ranges, based on their distribution. The chi-squared test was used to evaluate for differences in categorical data. Different linear models were used to evaluate the effects of the visual acuity (VA)/VF ratio on the evaluated parameters. All statistical analyses were performed using SPSS software (version 26.0.0; IBM SPSS Statistics, Chicago, IL, USA). Statistical significance was set at *P* < 0.05.

## Results

### Demographics of participants included in the analysis

All patients with RP had a definite genetic diagnosis and were divided into the following five groups based on their disease-causing genes: *CYP4V2* (17 patients, 27.42%), *EYS* (14 patients, 22.58%), *PRPH2* (7 patients, 11.29%), *RPGR* (11 patients, 17.74%), and *USH2A* (13 patients, 20.97%) (Table 1 and Supplementary Table 1). The mean age of the control group was 42.9 ± 3.3 years, and the individuals of each of the RP groups were in their third and fourth decades of life.

**Table 1:** Summary of the demographic and clinical characteristics of participants

RP(N = 62)							
		**Control** (N = 21)	***CYP4V2*** (N = 17)	***EYS*** (N = 14)	***PRPH2***(N = 7)	***RPGR***(N = 11)	***USH2A***(N = 13)
**Demographic**	**Gender, n (%)**						
	female	13 (61.9)	10 (58.8)	8 (57.1)	6 (85.7)	0 (0.0)	7 (53.8)
	male	8 (38.1)	7 (41.2)	6 (42.9)	1 (14.3)	11 (100.0)	6 (46.2)
	**Age, y**						
	Mean ± SD	42.9 ± 3.3	33.2 ± 3.8	39.1 ± 3.0	46.3 ± 6.9	31.1 ± 3.4	40.8 ± 2.8
	Median (IQR)	42.0(25.5)	38.0(11.5)	39.0 (16.3)	47.0 (27.0)	31.5 (14.3)	37.0 (14.5)
	Range	18.0–70.0	32.0–59.0	22.0–61.0	26.0–78.0	4.0–45.0	29.0–66.0
**Characteristic**	**BCVA (logMAR)**						
	Mean ± SD		1.4 ± 2.0	0.3 ± 0.2	0.6 ± 0.2	0.5 ± 0.1	0.8 ± 0.3
	Median (IQR)		0.6 (1.4)	0.2 (0.4)	0.4 (0.7)	0.5 (0.4)	0.3 (0.7)
	Range		0.0–4.0	0.0-1.5	0.1–1.7	0.2-1.0	0.0–4.0
	**Age at onset**						
	Mean ± SD		29.9 ± 2.5	18.4 ± 3.5	30.1 ± 11.3	6.6 ± 3.4	17.8 ± 4.1
	Median (IQR)		32.0(13.0)	21.0 (29.5)	44.0 (48.0)	0.0 (30.0)	17.0 (28.3)
	Range		9.0–45.0	0.0–36.0	0.0–73.0	0.0–30.0	0.0–38.0
	**Initial symptoms, n (%)**						
	Nyctalopia		12 (70.6)	13 (92.9)	6 (85.7)	11(100.0)	12 (92.3)
	Visual field loss		0 (0.0)	0 (0.0)	0 (0.0)	0 (0.0)	1 (7.7)
	Visual acuity loss		1 (5.9)	1 (7.1)	1 (14.3)	0 (0.0)	0 (0.0)
	Asymptomatic		4 (23.5)	0 (0.0)	0 (0.0)	0 (0.0)	0 (0.0)
	**Disease duration, y**						
	Mean ± SD		9.5 ± 2.2	20.7 ± 4.7	16.1 ± 4.9	24.5 ± 4.7	23.8 ± 5.1
	Median (IQR)		6.0 (18.0)	12.0 (30.8)	11.0 (25.0)	25.5 (28.3)	17.0 (33.2)
	Range		1.0–23.0	5.0–59.0	3.0–33.0	3.0–45.0	Jun-56
	**Visual field (MD), dB**						
	Mean ± SD		-23.7 ± 1.8	-19.3 ± 2.3	-24.7 ± 1.7	-21.3 ± 1.6	-26.2 ± 0.8
	Median (IQR)		-25.3(6.5)	-21.3 (5.6)	-23.1 (11.8)	-24.2 (7.3)	-25.2 (5.4)
	Range		-22.1	-23.7	-11.8	-21.2	-9.95

There seemed to be no significant differences in age and sex between the healthy control and patients with RP, except for the RPGR group, which consisted of all men influenced by the mode of inheritance.

There were no significant differences in BCVA among the RP groups (Bonferroni-corrected *P* = 0.242), although there was for the visual field (VF) mean deviation (MD) (-26.2 ± 0.8 dB) among the *USH2A* group compared to other RP groups (*CYP4V2*: -23.7 ± 1.8 dB, *P* = 0.047; *EYS*: -19.3 ± 2.3 dB, *P* = 0.011; *RPGR*: -21.3 ± 1.6 dB, *P* = 0.001) with the exception of the *PRPH2* group (-24.7 ± 1.7 dB, *P* = 0.391). The most common initial symptom in each group was nyctalopia, with a minority of patients demonstrating VF loss (7.7% in the *USH2*A group), decreased VA (5.9% in the *CYP4V2* group, 7.1% in the *EYS* group, 14.3% in the *PRPH2* group), and asymptomatic daily life (23.5% in the *CYP4V2* group). The age at onset (Bonferroni-corrected *P* = 0.041) and disease duration (Bonferroni-corrected *P* = 0.004) differed significantly among the RP groups, with the disease duration in the *RPGR* group being much longer than that in the *CYP4V2* group (*P* = 0.02).

### Retina and choroid blood volume

**Fig. 1:**
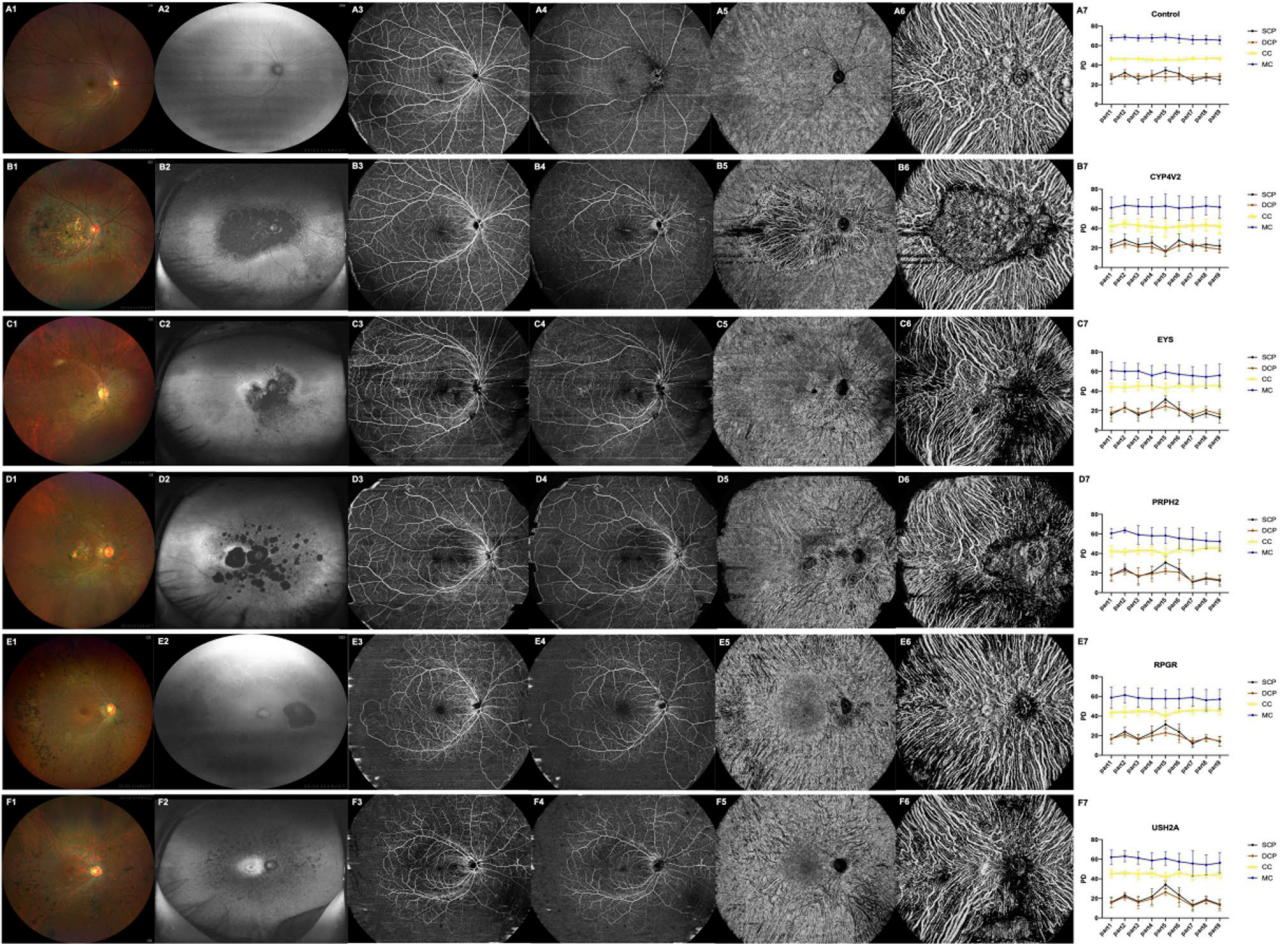
Typical color fundus photography, autofluorescence and SS-OCTA images of SCP, DCP, CC and MLC in each group with average PDs. **A1-F2**) color fundus photography and autofluorescence representative fundus in each group. **A3-F6**) the corresponding OCTA images of the retina and choroid. **A7-F7**) the average PDs of part1-9 of the four layers in each group

**Fig. 2:**
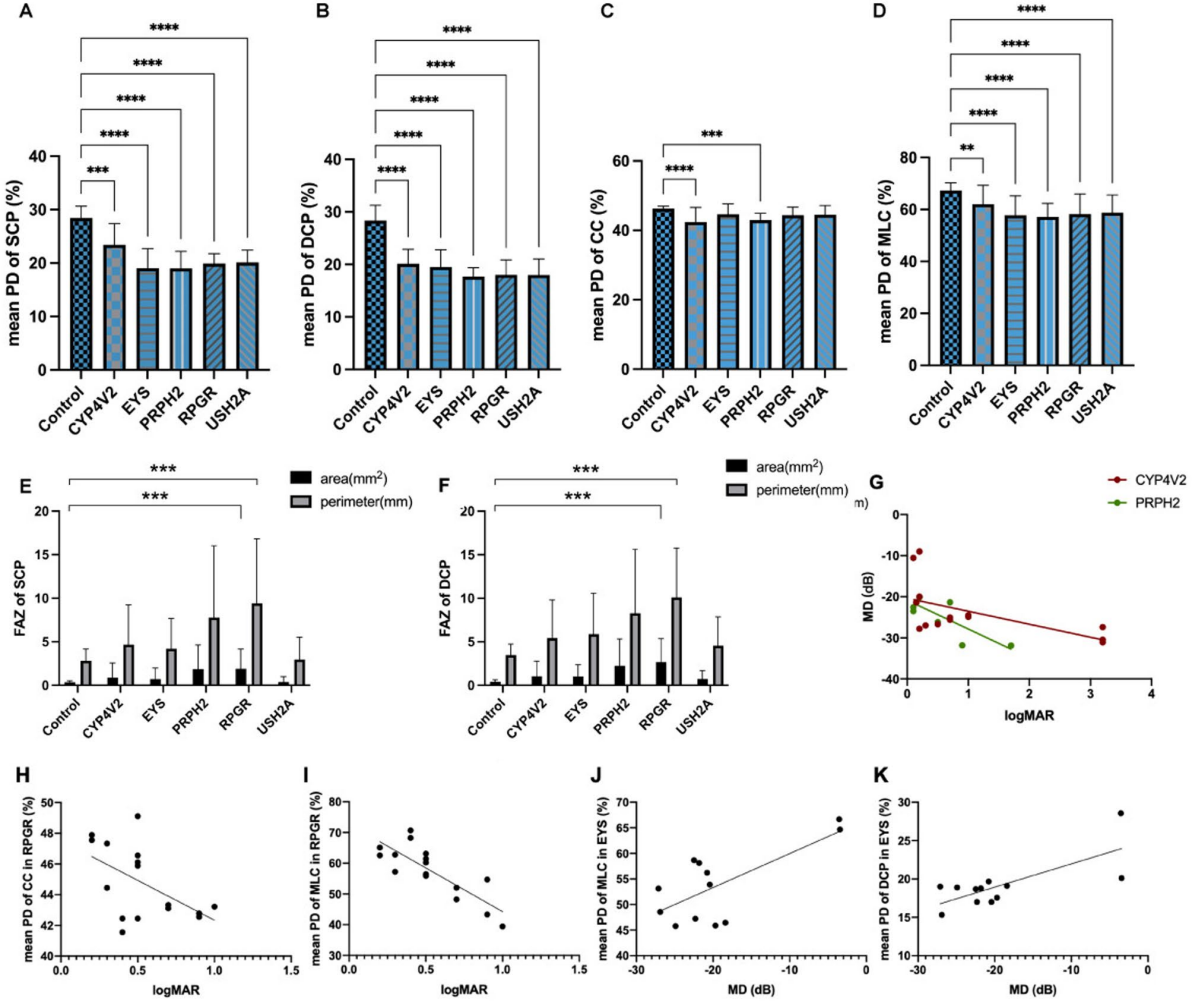
Mean PD of SCP, DCP, CC and MLC and FAZ size of each group and their linear relationship with retinal function. **A-D**) The fields in SCP, DCP, CC and MLC vascularity. **E-F**) The area and perimeter of FAZ of SCP and DCP. **G**) Liner relationships of VA and VF in *CYP4V2* and *PRPH2* groups (*P* < 0.05). **H-I**) Linear relationships between mean PD and BCVA of *RPGR* in CC and MLC (*P* < 0.05, *P* < 0.001). **J-K**) Linear relationship between mean PD and VF of EYS in MLC and DCP (*P* < 0.01, *P* < 0.01). * *P* < 0.05, ** *P* < 0.01, *** *P* < 0.001, **** *P* < 0.0001

An example of the SS-OCTA images of the SCP, DCP, CC, and MLC layers from each gene group demonstrated attenuation of PD (Figs. 1A3-F6). When quantified, the blood distribution of each layer decreased by varying degrees among the different groups (Figs. 2 A–D, *P* < 0.05), while the mean PD of the SCP decreased by 17.68% in the *CYP4V2*, 33.01% in the *EYS*, 33.15% in the *RPRH2*, 29.98% in the *RPGR*, and 29.28% in the *USH2A* groups. The mean PD of the DCP decreased 29.08% in the *CYP4V2*, 33.16% in the *EYS*, 37.62% in the *RPRH2*, 36.45% in the *RPGR*, and 36.56% in the *USH2A* groups. The mean PD in the CC decreased slightly in each group, 8.32% in the *CYP4V2*, 3.44% in the *EYS* (*P* = 0.09), 7.11% in the *RPRH2*, 4.08% in the *RPGR*, and 3.83% in the *USH2A* (*P* = 0.05) groups. The mean PD in the MLC decreased by 7.87% in the *CYP4V2*, 14.17% in the *EYS*, 15.12% in the *RPRH2*, 13.49% in the *RPGR* and 12.68% in the *USH2A* groups (Supplementary Table 2).

Following the comparison of the mean PDs, a detailed PD analysis of the nine parts of the fundus was conducted for each layer (Supplementary Table 2). In the control group, the PD of each area in the DCP, CC, and MLC was uniformly distributed across the layer, while the PD of the central part (part5) in the SCP was higher than that in the other areas (Figs. 1A7, *P* < 0.001). The distribution of PD among the nine parts showed a similar pattern across the *EYS*, *RPRH2*, *RPGR*, and *USH2A* groups, indicating significant hypoperfusion in the peripheral retina, particularly in inferior parts, and in the *EYS* group, the SPD was decimated at part7–9. However, for the *CYP4V2* group, a relatively large decrease was observed in the central part compared to the other parts (Figs. 1B7–F7, *P* < 0.001). These results were consistent with the fundus photography and fundus autofluorescence findings (Figs. 1A1*–*F2).

**Table 2:** Area and perimeter of FAZ in different groups

		Control	CYP4V2	EYS	PRPH2	RPGR	USH2A
**FAZ area** **in SCP (mm**^**2**^)		0.346 ± 0.029	0.886 ± 0.295	0.708 ± 0.263	1.873 ± 0.799	1.912 ± 0.507	0.379 ± 0.130
	R squared		0.005	0.476	0.040	0.005	0.028
	*P* value vs. Control		0.726	**0.0005**	0.580	0.784	0.484
**FAZ area** **in DCP (mm**^**2**^)		0.430 ± 0.340	1.047 ± 0.310	1.032 ± 0.262	2.251 ± 0.888	2.652 ± 0.606	0.728 ± 0.192
	R squared		0.184	0.000	0.582	0.000	0.007
	*P* value vs. Control		**0.023**	0.980	0.310	0.981	0.719
**FAZ perimeter in SCP (mm)**		2.827 ± 0.213	4.680 ± 0.805	4.202 ± 0.708	7.791 ± 2.377	9.412 ± 1.656	2.954 ± 0.538
	R squared		0.000	0.543	0.085	0.000	0.024
	*P* value vs. Control		0.951	**0.0001**	0.415	0.991	0.512
**FAZ perimeter in DCP (mm)**		3.480 ± 0.198	5.432 ± 0.772	5.872 ± 0.926	8.286 ± 2.116	10.10 ± 1.269	4.553 ± 0.663
	R squared		0.215	0.006	0.051	0.019	0.022
	*P* value vs. Control		**0.013**	0.718	0.530	0.583	0.504

Significantly lower PDs were observed in both the retinal and choroidal vessels of patients with RP compared to those of healthy controls (*P* < 0.001 for both). For the RP groups, SCP declined with different amplitudes through the whole field, except in part5 (posterior pole), in the *EYS*, *RPRH2*, *RPGR*, and *USH2A* groups, while only in part2 (12.71%, *P* = 0.028) and part5 (52.08%, *P* < 0.001) in the *CYP4V2* group (Supplementary Table 2). The DCP experienced a larger decrease than the SCP, showing attenuation in almost every part of nearly all RP groups, except for part5 of the *EYS* (12.01%, *P* = 0.074) and *USH2A* (5.64%, *P* = 1.000) groups (Supplementary Table 2). The PD of the CC in each RP group was slightly different from that of the retinal vessels, with parts affected in the *EYS* (part2: 6.15%, *P* = 0.041), *PRPH2* (part2: 10.10%, *P* < 0.001; part5: 14.11%, *P* = 0.008), *RPGR* (part5: 11.45%, *P* = 0.001), and *USH2A* (part5: 8.60%, *P* = 0.024; part8: 6.14%, *P* = 0.013) groups. However, PDs decreased in four fields, the inferior areas in particular, in the *CYP4V2* group (part1: 8.61%, *P* = 0.016; part7: 13.43%, *P* = 0.024; part8: 7.42%, *P* = 0.047; part9: 10.82%, *P* = 0.003). Patients with RP (*CYP4V2*, *EYS*, *PRPH2*, *RPGR*, and *USH2A*) also experienced extensive PD damage in the MLC, among which the *CYP4V2* group (part1: 9.95%, *P* = 0.008; part2: 7.60%, *P* = 0.016; part3: 7.82%, *P* = 0.007; part5: 10.55%, *P* = 0.009) was relatively less damaged (Supplementary Table 2).

Furthermore, the FAZ area and perimeter are larger in patients with RP than in controls (Figs. 2E–F; Table 2). owever, there was a large difference between individuals in the same group, with the FAZ showing statistically significant differences only in the *EYS* group in the SCP and in the *CYP4V2* group in the DCP.

### Correlation of OCTA parameters with retinal function analysis in RP

The relationship between VA and MD was analyzed in each RP group. A negative linear correlation between log MAR and MD, which reflects a positive association between BCVA and MD, was found in the *CYP4V2* (*P* = 0.037) and *PRPH2* (*P* < 0.001) groups, while no significant correlations were identified in the other RP groups (Fig. 2G). We then analyzed the correlations between the PD, BCVA, and MD. Although a linear dependence on mean PD was only identified in the *EYS* and *RPGR* groups (Fig. 2H–K), the PD in various parts was found to be associated with retinal function in each group (Table 3). A correlation between the PD and BCVA was identified in every part of the fundus in the *RPGR* group, especially in the MLC, while the PD and MD were not correlated. PD in the *USH2A* group showed a linear relationship with BCVA, but not with the MD (CC-part3: *r* = 0.446, *P* < 0.001; MLC-part2: *r* = 0.331, *P* = 0.004).

**Table 3:** Liner correlation analysis of PDs in different groups with BCVA/VF

		Layer	Part	Pearson *r*	*p* value
***CYP4V2***	**BCVA**	**MLC**	p2/ p4/ p5/ p6	0.270/ 0.173/ 0.248/ 0.235	0.005/ 0.028/ 0.008/ 0.009
	**VF**	**MLC**	p2/ p3/ p7/ p8/ p9	0.325/ 0.462 / 0.522/ 0.446/ 0.403	0.042/ 0.011/ 0.005 / 0.013/ 0.020
***EYS***	**BCVA**	**SCP**	p1/ p2/ p3/ p7	0.307/ 0.206/ 0.384/ 0.167/	0.003/ 0.020/ 0.001/ 0.038 /
		**MLC**	p1/ p3/ p7	0.175/ 0.183/ 0.172	0.034/ 0.030/ 0.035
	**VF**	**CC**	p1	0.435	0.020
		**MLC**	p4/ p6	0.380/ 0.405	0.033/ 0.026
***PRPH2***	**BCVA**	**CC**	p7	0.483	0.012
	**VF**	**SCP**	p2/ p7/ p5	0.681/ 0.589/ 0.530	0.012/ 0.026/ 0.041
***RPGR***	**BCVA**	**SCP**	p1	0.258	0.044
		**CC**	p4/ p5/ p7/ p8/	0.315/ 0.258/ 0.593 /0.324	0.024 /0.045 /<0.001/ 0.021
		**MLC**	p1/ p2/ p3/ p4/ p5/ p6/ p7/ p8/ p9	0.549/ 0.522/ 0.481/ 0.295/ 0.585/ 0.256/ 0.507/ 0.656/ 0.396	0.001/ 0.002/ 0.003/ 0.030/ <0.001/ 0.046/ 0.002/ < 0.001/ 0.009
	**VF**		-	-	-
***USH2A***	**BCVA**	**CC**	p3	0.446	< 0.001
		**MLC**	p2	0.331	0.004
	**VF**		-	-	-

The MD was mainly related to the *CYP4V2* mutation that causes hypoperfusion in the MLC. Part7 of the CC in the *PRPH2* group was linearly related to the BCVA (CC-part7, *r* = 0.483, *P* = 0.012). Next, we analyzed CVI in RP patients and healthy controls, and their correlation with retinal function. Detailed information is provided in Supplementary Table 3. The CVI was extensively reduced in the RP group compared to healthy individuals. The CVI of the superior parts (part1–3) were significantly reduced by varying degrees in each group, ranging from 14.42% (*USH2A*) to 23.42% (*CYP4V2*). In part4–6, an obvious decrease in the CVI was noted, albeit with large fluctuations between different patients within the same group, ranging from 16.01% (*USH2A*) to 29.61% (*EYS*). Comparison of the CVI in the inferior fields (part7–9) between the RP and control groups, however, showed no statistical significance in the *CYP4V2* (10.02%, *P* = 1.000; 7.59%, *P* = 1.000; 16.31%, *P* = 0.199) and *RPGR* (21.46%, *P* = 0.092; 19.90%, *P* = 0.166; 18.04%, *P* = 0.206) groups. In contrast, the CVI of the *EYS* group (25.28%, *P* = 0.004; 28.06%, *P* = 0.004; 26.25%, *P* = 0.006) significantly decreased in the inferior areas (part7–9). A linear correlation was also observed between the CVI and retinal function parameters (BCVA and MD value), demonstrating an association between the CVI and BCVA, especially in the *RPGR* group (Supplementary Table 3).

## Discussion

Several studies have evaluated the relationship between ocular volume and RP, albeit with contrasting conclusions [12–14]. These findings often focused on a single gene mutation or did not distinguish between the genotypes associated with RP [15]. To explore the features and vasculature damage caused by different genes, we grouped 124 eyes from 62 patients and described the PD of nine fields in the SCP, DCP, CC, and MLC layers. We evaluated retinal and choroidal vascularity in an extensive area by dividing the vasculature into different fields, and determined the following: (1) defects were observed in every RP group, regardless of the genotype, in the SCP, DCP, CC, and MLC layers to varying degrees; (2) patients with different genotypes showed different damage patterns in the SCP, DCP, CC, and MLC vasculature according to the PDs in the posterior pole and different perifundus regions in eyes with RP, with the *CYP4V2* mutation in particular demonstrating opposite variation trends when compared with other RP groups; (3) FAZ area and perimeter of the SCP and DCP in the *CYP4V2* and *EYS* groups, respectively, were significantly larger than those in healthy individuals, with large intragroup differences; (4) BCVA and MD were linearly correlated with mean PD only in the *EYS* and *RPGR* groups, although the PDs of different parts were associated with retinal function in each group except the *RPGR* and *USH2A* groups, and PD in every part was not correlated with the MD value; and (5) the CVI decreased by variable degrees with different patterns among the RP groups, and was associated with the BCVA or MD.

Although strong evidence suggests that vascular dysfunction causes photoreceptor (PR) loss, vascular dysfunction in RP may have secondary effects, serving as a diagnostic, prognostic, and therapeutic tool in the clinical setting [16]. Some studies have provided evidence that vascular dysfunction is not significant in early-stage RP, suggesting that the development of vascular dysfunction is directly dependent on the progression of PR degeneration [17].

Previous studies have reported that superficial and deep capillary plexus vessel density in the macula and optic nerve head was significantly lower in patients with RP than in healthy controls, with similar results for superficial and deep capillary plexus vessel density in the parafoveal region [18, 19]. Other studies obtained different conclusions regarding lower flow density in the foveal and parafoveal superficial capillary plexuses and parafoveal deep capillary plexus, but not in the foveal deep capillary plexus, in eyes with RP [13, 14]. Many researchers have confirmed choroidal and retinal vessel alterations in patients with RP using small-field OCTA and have recognized changes in ocular blood flow and retinal vascularization as clinical features of RP. Moreover, a fully recognized hypothesis regarding the role of retinal vascular alterations in the pathogenesis of RP has been proposed. Systolic and end-diastolic blood flow velocities in the central retinal artery (CRA) and short posterior ciliary arteries (SPCA) were found to exhibit a direct correlation with the amplitudes of full-field electroretinography (ERG) a-waves and b-waves, respectively [20]. Wide-field OCTA (12 × 12 mm) has been performed to assess retinal vascular and choroidal alterations in patients with RP by evaluating vessel length and vessel density, which were reduced in both the central and peripheral retinas [21]. 

In this cross-sectional study, we demonstrated substantial reductions in PD in the SCP, DCP, CC, and MLC in RP groups, with significant differences between each group, especially in the *CYP4V2* group, which showed a unique pattern. These results highlight the practical importance of ascertaining the genotype involved in RP, as various mutations can cause disparate anatomical defects. The FAZ area and perimeter are reported to be larger in patients with RP than in controls in previous research [12, 22, 23]. Consistent with the previous research, the FAZ area was much larger in eyes with RP than in the healthy controls in both the SCP and DCP in our study, indicating the role of FAZ disruption in RP. Interestingly, BCVA and VF were linearly dependent in the *CYP4V2* and *PRPH2* groups, suggesting that distinct mutations can lead to dissimilar functional alterations.

These results showed that the largely decreased PD of the central part in the SCP, together with fundus photography and fundus autofluorescence findings, can help clinicians distinguish RP patients carrying mutations in the *CYP4V2* gene. Significantly lower PDs, larger FAZ areas and decreased CVI can also be used as biomarkers in indicating abnormal retinal function in RP patients.

Our study also showed some limitations. For example, the ophthalmic baseline data of the control group, such as BCVA and VF, are incomplete, which might affect the interpretation of the retinal and choroidal perfusion patterns of inherited retinal diseases to some extent. While incomplete, the available control data still allowed us to conclude that patients with RP had decreased PDs in the retina and choroid. Another limitation was the non-application of statistical models accounting for intra-subject correlation in our study. However, given the study’s primary objective to explore between-group differences, the impact of this limitation on the core findings was considered minimal.

In summary, as a non-invasive imaging tool to identify and quantify ocular vasculature, OCTA has emerged as a powerful and useful technique to support the diagnosis and prognosis of retinitis pigmentosa to some extent. OCTA imaging can assist clinicians in early detection of RP, determining the subgroup and progression of RP, and, at the same time, developing personalized drug intervention plans for different RP patients and evaluating their treatment efficiency. In addition, OCTA has the potential to be applied in more eye diseases involving diabetic retinopathy, age-related macular degeneration and optic nerve disorders.

## Electronic supplementary material

Below is the link to the electronic supplementary material.

Supplementary Material 1

## Data Availability

Data is provided within the manuscript or supplementary information files.

## Abbreviations

SS-OCTA: Swept-source optical coherence tomography angiography
OCT: Optical coherence tomography
PD: Perfusion density
SCP: Superficial capillary plexus
DCP: Deep capillary plexus
CC: Choriocapillaris
MLC: Medium-large choroidal vessel layer
CVI: Choroidal vascularity index
FAZ: Foveal avascular zone
RP: Retinitis pigmentosa
ERG: Electroretinography
VF: Visual field
BCVA: Best-corrected visual acuity
VA: Visual acuity
FL: Fixation loss
FP: False-positive
IOP: Intraocular pressure
IPL: Inner plexiform layer
OPL: Outer plexiform layer
WGS: Whole-genome sequencing
WES: Whole-exome sequencing
