# Supplementary material for: Genotype-specific retinal and choroidal perfusion patterns in inherited retinal diseases: an SS-OCTA analysis
Source: Int J Retina Vitreous. 2025 Jul 23;11:82. doi: 10.1186/s40942-025-00706-0 (PMC12288356; doi:10.1186/s40942-025-00706-0)
Supplement: Supplementary file 1 — Supplementary Material 1 [file 40942_2025_706_MOESM1_ESM.docx]

Supplementary table 1. Mutation information of RP patients

| **group** | **No.** | **inheritance** | **Nucleotide alteration** | **Amino acid change** | **heterozygosity** | |
| --- | --- | --- | --- | --- | --- | --- |
| ***CYP4V2*** | 1 | AR | c.1091-2A>G | - | Hom |  |
|  | 2 | AR | c.1091-2A>G | - | Het |  |
|  |  | AR | c.802-8_810delTCATACAGGTCATCGCTinsGC | - | Het |  |
|  | 3 | AR | c.802-8_807delTCATACAGGTCATC | - | Het |  |
|  |  | AR | c.810delT | p.Ala270Alafs7 | Het |  |
|  |  | AR | c.992A>C | p.His331Pro\|p.H331P | Het |  |
|  | 4 | AR | c.802-8_810delTCATACAGGTCATCGCTinsGC |  | Hom |  |
|  | 5 | AR | c.802-8_810delTCATACAGGTCATCGCTinsGC | - | Hom |  |
|  | 6 | AR | c.802-8_810delTCATACAGGTCATCGCTinsGC |  | Hom |  |
|  | 7 | AR | c.992A>C | p.His331Pro | Het |  |
|  |  | AR | c.802-8_810delTCATACAGGTCATCGCTinsGC |  | Het |  |
|  | 8 | AR | c.802-8_807delTCATACAGGTCATC | - | Hom |  |
|  |  | AR | c.810delT | p.Glu271Asnfs*6 | Hom |  |
|  | 9 | AR | c.802-8_807delTCATACAGGTCATC | - | Hom |  |
|  |  | AR | c.810delT | p.Glu271Asnfs*6 | Hom |  |
|  | 10 | AR | c.1229G>T | p.Gly410Val | Het |  |
|  |  | AR | c.1091-2A>G | - | Het |  |
|  | 11 | AR | c.1091-2A>G | - | Het |  |
|  |  | AR | c.802-8_810delTCATACAGGTCATCGCTinsGC | - | Het |  |
|  | 12 | AR | c.802-8_807delTCATACAGGTCATC | - | Het |  |
|  |  | AR | c.810delT | p.Glu271Asnfs*6 | Het |  |
|  |  | AR | c.1091-2A>G | - | Het |  |
|  | 13 | AR | c.802-8_807delTCATACAGGTCATC | - | Hom |  |
|  |  | AR | c.810delT | p.Glu271Asnfs*6 | Hom |  |
|  | 14 | AR | c.802-8_807delTCATACAGGTCATC | - | Het |  |
|  |  | AR | c.810delT | p.Glu271Asnfs*6 | Het |  |
|  |  | AR | c.1198C>T | p.Arg400Cys\|p.R400C | Het |  |
|  | 15 | AR | c.802-8_807delTCATACAGGTCATC | - | Hom |  |
|  |  | AR | c.810delT | p.Glu271Asnfs*6 | Hom |  |
|  | 16 | AR | c.992A>C | p.His331Pro | Het |  |
|  |  | AR | c.237G>T | p.Glu79Asp | Het |  |
|  | 17 | AR | c.802- 8_810delTCAT ACAGGTCAT CGCTinsGC | - | Hom |  |
| ***EYS*** | 18 | AR | c.3359A>G | (p.Asn1120Ser) | Het |  |
|  |  | AR | c.1185- 6T>G | - | Het |  |
|  |  | AR | c.971C>A | (p .Ser324Tyr) | Het |  |
|  | 19 | AR | c.6416G>A | p.Cys2139Tyr | Hom |  |
|  | 20 | AR | c.6416G>A | (p.Cys2139Tyr) | Het |  |
|  |  | AR | c.7492G>C | p.Ala2498Pro) | Het |  |
|  | 21 | AR | c.5677_5681del | p.Tyr1893Argfs12\|p.Y1893Rfs12 | Het |  |
|  |  | AR | c.992C>T | p.Thr331Ile\|p.T331I | Het |  |
|  | 22 | AR | c.2486delT | p.Ile829Thrfs*39 | Het |  |
|  |  | AR | c.8096T>C | p.Phe2699Ser | Het |  |
|  | 23 | AR | c.8159T>C | p.Phe2720Ser\|p.F2720S | Het |  |
|  |  | AR | c.2486del | p.Ile829Thrfs39\|p.I829Tfs39 | Het |  |
|  | 24 | AR | c.7630C>T | p.Leu2544Phe\|p.L2544F | Het |  |
|  |  | AR | c.5902G>A | p.Gly1968Arg\|p.G1968R | Het |  |
|  | 25 | AR | c.2380C>T | p.Arg794* | Het |  |
|  |  | AR | EX15 Del | - | Het |  |
|  | 26 | AR | c.7207T>C | p.Ser2403Pro | Hom |  |
|  | 27 | AR | c.8170G>T | p.Glu2724*\|p.E2724* | Het |  |
|  |  | AR | c.9193T>C | p.Trp3065Arg\|p.W3065R | Het |  |
|  | 28 | AR | c.8805C>A | p.Tyr2935* | Het |  |
|  |  | AR | c.8970_897 1insGT | p.Thr29 91Valfs*5 | Het |  |
|  | 29 | AR | c.6416G>A | p.Cys2139Tyr | Het |  |
|  |  | AR | c.9019G>T | p.Asp3007Tyr | Het |  |
|  | 30 | AR | c.6416G>A | p.Cys2139Tyr | Het |  |
|  |  | AR | c.8012T>A | p.Leu2671* | Het |  |
|  |  | AR | c.1185- 6T>G | - | Het |  |
|  | 31 | AR | c.5644+5G>A | - | Het |  |
|  |  | AR | c.5305delG | (p.Asp1769Thrfs*9) | Het |  |
| ***PRPH2*** | 32 | AD | c.657_662del | p.Arg220_Pro221del\|p.R220_P221del | Het |  |
|  | 33 | AD | c.631T>C | p.Phe211Leu | Het |  |
|  | 34 | AD | c.595A>G | p.Asn199Asp | Het |  |
|  | 35 | AD | c.798_799insT | p.Val267Cysfs34\|p.V267Cfs34 | Het |  |
|  | 36 | AD | c.508G>A | p.Gly170Ser | Het |  |
|  | 37 | AD | c.631T>C | p.Phe211Leu | Het |  |
|  | 38 | AD | c.631T>C | p.Phe211Leu | Het |  |
| ***RPGR*** | 39 | XL | c.1512_1513delCA | p.Ile505Hisfs*7 | Hemi |  |
|  | 40 | XL | c.2129delA | p.Gln710Argfs*105 | Hemi |  |
|  | 41 | XL | c.595_599dupTATTA | p.His201Ilefs*7 | Hemi |  |
|  | 42 | XL | c.3027_3028delGG | p.Glu1010Glyfs*68 | Hemi |  |
|  | 43 | XL | c.2405_2406delAG | p.Glu802Glyfs*32 | Hemi |  |
|  | 44 | XL | c.2149C>T | p.Gln717*\|p.Q717* | Hemi |  |
|  | 45 | XL | c.469+2T>C | - | Hemi |  |
|  | 46 | XL | c.2227A>C | p.Lys743Gln | Hemi |  |
|  | 47 | XL | c.2215G>T | p.Glu739*\|p.E739* | Hemi |  |
|  | 48 | XL | c.2395G>T | p.Glu799*\|p.E799* | Hemi |  |
|  | 49 | XL | c.2236_223 7delGA | p.Glu7 46Argfs*23 | Hemi |  |
| ***USH2A*** | 50 | AR | c.15104_15105delCA | (p.Thr5035Argfs*142) | Het |  |
|  |  | AR | c.8254G>A | (p.Gly2752Arg) | Het |  |
|  | 51 | AR | c.11156G>A | (p.Arg3719His) | Het |  |
|  |  | AR | c.9958G>T | (p.G ly3320Cys) | Het |  |
|  |  | AR | c.8284C>G | (p.P ro2762Ala) | Het |  |
|  | 52 | AR | c.2802T>G | (p.Cys934Trp) | Het |  |
|  |  | AR | c.4094T>A | (p.Met1365Lys) | Het |  |
|  | 53 | AR | c.4576G>A | p.Gly1526Arg\|p.G1526R | Het |  |
|  |  | AR | c.3314A>C | p.Tyr1105Ser\|p.Y1105S | Het |  |
|  | 54 | AR | c.15017C>T | p.Thr5006Met\|p.T5006M | Het |  |
|  |  | AR | c.1143+2T>C | _ | Het |  |
|  | 55 | AR | c.5204G>A | p.Gly1735Glu\|p.G1735E | Het |  |
|  |  | AR | c.2802T>G | p.Cys934Trp\|p.C934W | Het |  |
|  | 56 | AR | c.11806A>C | p.Thr3936Pro\|p.T3936P | Het |  |
|  |  | AR | c.6967C>T | p.Arg2323*\|p.R2323* | Het |  |
|  | 57 | AR | c.11389+3A>T | - | Het |  |
|  |  | AR | c.1551-15T>A | - | Het |  |
|  | 58 | AR | c.2802T>G | (p.Cys934Trp) | Het |  |
|  |  | AR | c.11549-1G>T | - | Het |  |
|  | 59 | AR | c.5836C>T | p.Arg1946Ter | Het |  |
|  |  | AR | c.2802T>G | p.Cys934Trp | Het |  |
|  | 60 | AR | c.8559-2A>G | - | Het |  |
|  |  | AR | c.12015A>G | p. (Arg4005=) | Het |  |
|  | 61 | AR | c.15178T>C | p. Ser5060Pro | Het |  |
|  |  | AR | c.5237C>T | p.T hr1746Ile | Het |  |
|  |  | AR | c.5051C>T | p.Pr o1684Leu | Het |  |
|  | 62 | AR | c.8559-2A>G | - | Het |  |
|  |  | AR | c.14404T>C | p.Ser4802Pro\|p.S4802P | Het |  |

Supplementary table 2. PDs of different groups and its amplitude

|  |  | **Control** | ***CYP4V2*** | ***EYS*** | ***PRPH2*** | ***RPGR*** | ***USH2A*** | **Amplitude（%）** | ***p* value vs control** |
| --- | --- | --- | --- | --- | --- | --- | --- | --- | --- |
| **SCP** | **Part1** | 25.925 ± 0.772 | 22.750 ± 1.093 | 16.115 ± 1.426 | 17.917 ± 1.616 | 16.524 ± 1.037 | 16.000 ± 1.103 | 12.25/ 37.84/ 30.89/ 36.26/ 38.28 | 0.686/ **<0.001/0.005/ <0.001/ <0.001** |
|  | **Part2** | 32.325 ± 0.497 | 28.218 ± 1.072 | 23.192 ± 0.995 | 24.333 ± 1.389 | 24.048 ± 1.066 | 22.960 ± 0.615 | 12.71/ 28.25/ 24.72/ 25.61/ 28.97 | **0.028**/ **<0.001/ <0.001/ <0.001/ <0.001** |
|  | **Part3** | 25.500 ± 0.693 | 23.281 ± 1.070 | 15.654 ± 1.230 | 16.500 ± 2.563 | 16.667 ±1.029 | 16.880 ± 0.847 | 8.70/ 38.61/ 35.29/ 34.64/ 33.80 | 1.000/ **<0.001/ 0.002/ <0.001/ <0.001** |
|  | **Part4** | 29.350 ± 0.836 | 25.219 ± 1.111 | 20.731 ± 1.524 | 20.667 ± 1.818 | 23.000 ± 1.532 | 21.920 ± 1.561 | 14.07/ 29.37/ 29.58/ 21.64/ 25.32 | 0.147/ **<0.001/ 0.003/ 0.016/ 0.002** |
|  | **Part5** | 34.825 ± 0.467 | 16.688 ± 0.939 | 31.346 ± 0.736 | 30.667 ± 1.781 | 31.429 ± 0.815 | 34.040 ± 0.81 | 52.08/ 9.99/ 11.94/ 9.75/ 2.25 | **<0.001/** 0.070/ 0.766/ 0.162/ 1.000 |
|  | **Part6** | 31.350 ± 0.959 | 27.656 ± 1.167 | 21.577 ± 1.407 | 24.000 ± 2.796 | 23.905 ± 1.707 | 23.480 ± 1.541 | 11.78/ 31.17/ 23.44/ 23.75/ 25.10 | 0.691/ **<0.001**/ 0.167/ **0.011/ 0.002** |
|  | **Part7** | 24.025 ± 0.536 | 21.594 ± 0.812 | 12.500 ± 0.876 | 10.667 ± 1.798 | 11.857 ± 0.900 | 13.200 ± 1.042 | 10.12/ 47.97/ 55.60/ 50.65/ 45.06 | 1.000/ **<0.001**/ **<0.001**/ **<0.001**/ **<0.001** |
|  | **Part8** | 27.750 ± 0.632 | 23.594 ± 1.195 | 17.231 ± 0.795 | 14.167 ± 1.595 | 17.857 ± 0.670 | 18.840 ± 0.756 | 14.98/ 37.91/ 48.95/ 35.65/ 32.11 | 0.123/ **<0.001/ <0.001**/ **<0.001**/ **<0.001** |
|  | **Part9** | 25.500 ±0.713 | 21.813 ± 1.135 | 13.154 ± 1.135 | 12.250 ± 1.666 | 14.000 ± 1.019 | 13.760 ± 0.942 | 14.46/ 48.42/ 51.96/ 45.10/ 46.04 | 1.000/ **<0.001**/ **<0.001**/ **<0.001**/ **<0.001** |
| **DCP** | **Part1** | 28.475 ± 2.924 | 20.628 ± 6.803 | 18.038 ± 5.148 | 18.000 ± 6.608 | 16.714 ± 4.661 | 15.480 ± 4.809 | 27.56/ 36.65/ 36.79/ 41.30/ 45.64 | **<0.001**/ **<0.001**/ **<0.001**/ **<0.001**/ **<0.001** |
|  | **Part2** | 28.675 ± 2.893 | 24.128 ± 4.398 | 22.731 ± 3.020 | 22.250 ± 3.897 | 21.143 ± 3.328 | 21.480 ± 3.164 | 15.86/ 20.73/ 22.41/ 26.27/ 25.09 | **0.002**/ **<0.001**/ **<0.001**/ **<0.001**/ **<0.001** |
|  | **Part3** | 28.625 ± 2.808 | 20.609 ± 7.094 | 17.385 ± 4.279 | 17.333 ± 7.706 | 15.952 ± 4.582 | 15.920 ± 6.318 | 28.00/ 39.27/ 39.45/ 44.27/ 44.38 | **<0.001**/ **<0.001**/ **<0.001**/ **<0.001**/ **<0.001** |
|  | **Part4** | 28.650 ± 3.005 | 22.365 ± 6.883 | 20.346 ± 6.348 | 19.083 ± 7.216 | 19.905 ± 5.442 | 19.560 ± 7.300 | 21.94/ 28.98/ 33.39/ 30.52/ 31.73 | **<0.001**/ **<0.001**/ **<0.001**/ **<0.001**/ **<0.001** |
|  | **Part5** | 27.850 ± 3.664 | 23.503 ± 5.716 | 24.480 ± 3.775 | 21.917 ± 2.753 | 23.048 ± 3.184 | 26.280 ± 3.256 | 15.61/ 12.10/ 21.30/ 17.24/ 5.64 | **<0.001**/ 0.074/ **0.002**/ **0.002**/ 1.000 |
|  | **Part6** | 28.850 ± 3.175 | 22.968 ± 6.959 | 20.692 ± 5.319 | 20.833 ± 8.335 | 19.952 ± 6.911 | 20.000 ± 7.970 | 20.39/ 28.28/ 27.79/ 30.84/ 30.68 | **<0.001**/ **<0.001**/ **0.004**/ **<0.001**/ **<0.001** |
|  | **Part7** | 27.175 ± 3.240 | 17.513 ± 5.732 | 15.538 ± 4.986 | 11.333 ± 3.325 | 13.667 ± 4.357 | 12.200 ± 5.381 | 35.55/ 42.82/ 58.30/ 49.71/ 55.11 | **<0.001**/ **<0.001**/ **<0.001**/ **<0.001**/ **<0.001** |
|  | **Part8** | 28.375 ± 2.680 | 21.141 ± 7.390 | 20.154 ± 3.968 | 15.083 ± 4.329 | 17.238 ± 3.544 | 17.800 ± 3.510 | 25.49/ 28.97/ 46.84/ 39.25/ 37.27 | **<0.001**/ **<0.001**/ **<0.001**/ **<0.001**/ **<0.001** |
|  | **Part9** | 28.375 ± 3.128 | 18.936 ± 1.797 | 16.423 ± 4.924 | 13.250 ± 4.585 | 14.476 ± 4.697 | 13.120 ± 5.552 | 33.27/ 42.12/ 53.30/ 48.98/ 53.76 | **<0.001**/ **<0.001**/ **<0.001**/ **<0.001**/ **<0.001** |
| **CC** | **Part1** | 42.313 ± 6.317 | 42.313 ± 6.317 | 44.308 ± 4.093 | 42.313 ±6.317 | 42.313 ±6.317 | 45.080 ± 3.730 | 8.61/ 4.30/ 9.11/ 6.10/ 2.63 | **0.016**/ 0.979/ 0.056/ 0.129/ 1.000 |
|  | **Part2** | 46.300 ± 1.584 | 44.750 ± 3.800 | 43.500 ± 4.050 | 42.083 ± 5.775 | 43.476 ± 4.294 | 45.680 ± 2.111 | 3.45/ 6.15/ 10.10/ 5.79/ 1.45 | 0.849/ **0.041**/ **<0.001**/ 1.000/ 1.000 |
|  | **Part3** | 46.350 ± 0.853 | 43.188 ± 5.763 | 45.077 ± 4.437 | 41.667 ± 3.257 | 43.667 ± 5.195 | 44.680 ± 4.628 | 7.02/ 2.96/ 7.43/ 3.43/ 3.81 | 1.000/ 1.000/ 0.058/ 1.000/ 1.000 |
|  | **Part4** | 46.450 ± 1.377 | 41.438 ± 6.344 | 45.308 ± 3.969 | 43.000 ± 3.908 | 44.857 ± 3.413 | 45.640 ± 3.273 | 9.38/ 0.91/ 5.78/ 2.21/ 0.19 | 0.036/ 1.000/ 0.883/ 1.000/ 1.000 |
|  | **Part5** | 45.725 ± 1.162 | 42.781 ± 5.170 | 42.654 ± 5.061 | 43.083 ± 4.316 | 44.714 ± 2.639 | 41.680 ± 4.388 | 6.18/ 6.46/ 14.11/ 11.45/ 8.60 | 0.577/ 0.751/ **0.008/ 0.001/ 0.024** |
|  | **Part6** | 45.600 ± 0.889 | 41.781 ± 6.097 | 45.885 ± 2.966 | 39.167 ± 6.351 | 40.381 ± 3.811 | 45.920 ± 2.741 | 8.47/ -0.51/ 3.80/ 1.74/ -0.59 | 0.271/ 1.000/ 1.000/ 1.000/ 1.000 |
|  | **Part7** | 45.650 ± 0.989 | 40.344 ± 8.816 | 44.154 ± 3.880 | 43.917 ± 3.753 | 44.857 ± 2.474 | 43.320 ± 5.026 | 13.43/ 5.25/ 7.55/ 2.21/ 7.04 | **0.024**/ 0.507/ 0.306/ 1.000/ 0.189 |
|  | **Part8** | 46.600 ± 1.578 | 43.281 ± 5.762 | 45.308 ± 2.700 | 43.083 ± 4.400 | 45.571 ± 3.824 | 43.880 ± 3.525 | 7.42/ 3.09/ 2.50/ 0.59/ 6.14 | **0.047**/ 0.507/ 1.000/ 1.000/ **0.013** |
|  | **Part9** | 46.750 ± 1.067 | 41.938 ± 7.119 | 45.923 ± 3.782 | 45.583 ± 1.975 | 46.476 ± 2.260 | 44.640 ± 3.393 | 10.82/ 2.34/ 3.77/ 3.39/ 5.07 | **0.003**/ 1.000/ 1.000/ 0.891/ 0.141 |
| **MLC** | **Part1** | 67.775 ± 3.174 | 61.031 ± 10.801 | 60.962 ± 8.478 | 60.500 ± 4.787 | 58.810 ± 10.509 | 61.960 ± 7.258 | 9.95/ 10.05/ 10.73/ 13.23/ 8.58 | **0.008/ 0.001/ 0.001/ 0.001/ 0.007** |
|  | **Part2** | 68.725 ± 2.302 | 63.500 ± 8.775 | 60.038 ± 8.487 | 63.500 ± 2.432 | 61.333 ± 8.079 | 63.080 ± 5.614 | 7.60/ 12.64/ 7.60/ 10.76/ 8.21 | **0.016**/ **<0.001**/ **0.005**/ **<0.001**/ **0.001** |
|  | **Part3** | 67.700 ± 3.059 | 62.406 ± 7.327 | 60.423 ± 7.722 | 59.000 ± 9.354 | 58.571 ± 9.515 | 61.200 ± 6.177 | 7.82/ 10.75/ 12.85/ 13.48/ 9.6 | **0.007/ <0.001/ 0.006/ <0.001/ 0.001** |
|  | **Part4** | 67.825 ± 3.680 | 61.813 ± 10.245 | 55.885 ± 10.032 | 58.000 ± 8.052 | 57.714 ± 10.273 | 58.600 ± 8.030 | 8.86/ 17.60/ 14.49/ 14.91/ 13.6 | 0.091/ **<0.001/ 0.002/ <0.001/ <0.001** |
|  | **Part5** | 68.750 ± 3.152 | 61.500 ± 10.700 | 59.538 ± 6.710 | 58.250 ± 7.896 | 57.619 ± 8.952 | 60.680 ± 6.656 | 10.55/ 13.40/ 15.27/ 16.19/ 11.74 | **0.009**/ **<0.001/ <0.001/ <0.001/ <0.001** |
|  | **Part6** | 67.375 ± 4.380 | 60.656 ± 12.571 | 57.000 ± 9.127 | 55.500 ± 9.811 | 57.857 ± 9.790 | 57.480 ± 8.401 | 9.97/ 15.40/ 17.63/ 14.13/ 14.69 | 0.227/ **<0.001/ 0.001/ 0.001/ <0.001** |
|  | **Part7** | 66.050 ± 4.421 | 62.813 ± 12.027 | 55.769 ± 8.988 | 54.417 ± 11.665 | 59.000 ± 8.821 | 55.600 ± 12.974 | 4.90/ 15.57/ 17.61/ 10.67/ 15.82 | 1.000/ **<0.001/ 0.016/** 0.067**/ 0.011** |
|  | **Part8** | 66.200 ± 4.136 | 62.906 ± 9.207 | 54.500 ± 11.500 | 52.917 ± 7.826 | 56.143 ± 10.656 | 54.160 ± 10.224 | 4.98/ 17.67/ 20.07/ 15.19/ 18.19 | 1.000/ **<0.001/ <0.001/ 0.001/ <0.001** |
|  | **Part9** | 65.575 ± 4.177 | 61.625 ± 10.882 | 56.038 ± 11.288 | 52.250 ± 9.479 | 57.238 ± 9.724 | 56.320 ± 10.464 | 6.02/ 14.54/ 20.32/ 12.71/ 14.11 | 1.000/ **0.003**/ **<0.001/ 0.014/ 0.002** |

Supplementary table 3. CVI of different groups and the correlation analysis with BCVA/VF

|  | **Control** | ***CYP4V2*** | ***EYS*** | ***PRPH2*** | ***RPGR*** | ***USH2A*** | ***p* value vs control and descend amplitudes** | **p value of the Pearson test with BCVA** | **p value of the Pearson test with VF** |
| --- | --- | --- | --- | --- | --- | --- | --- | --- | --- |
| **CVI p1** | 34.075 ± 0.705 | 26.094 ± 1.414 | 28.577 ± 1.508 | 28.500 ± 1.041 | 28.333 ± 1.875 | 29.160 ± 1.355 | **<0.001** (23.42%)/ **0.020** (16.13%)/ **0.020** (16.36%)**/ 0.090** (16.85%)**/ 0.029**(14.42%) | 0.659/ 0.456/ 0.366/ **0.020**/ 0.172 | 0.524/ 0.782/ 0.876/ -/ 0.600 |
| **CVI p2** | 34.150 ± 0.547 | 28.906 ± 1.240 | 26.308 ±1.516 | 28.417 ± 0.733 | 28.762 ± 1.790 | 28.260 ±1.152 | **0.007** (15.36%)**/ <0.001** (22.96%)**/ 0.010** (16.79%)**/ 0.035** (15.78%)**/ 0.001** (17.52%) | **0.014/** 0.480/ 0.552/ **0.001**/ 0.575 | 0.115/ 0.495/ 0.854/ -/ 0.439 |
| **CVI p3** | 33.575 ± 0.637 | 27.313 ± 0.949 | 25.923 ± 1.483 | 26.000 ± 2.256 | 26.381 ± 1.680 | 27.120 ± 1.201 | **<0.001** (18.65%)**/ <0.001** (22.79%)**/ 0.016** (22.56%)**/ 0.020** (21.43%)**/ <0.001** (19.23%) | 0.396/ 0.865/ 0.933/ **0.008**/ **<0.001** | **0.038**/ 0.921/ 0.849/ -/ 0.635 |
| **CVI p4** | 31.200 ± 0.817 | 24.469 ± 1.304 | 22.000 ± 1.621 | 24.500 ± 1.929 | 24.238 ± 1.623 | 23.800 ± 1.342 | **0.002** (21.57%)**/ <0.001** (29.49%)**/** 0.116 (21.47%)**/ 0.011** (22.31%)**/ 0.001** (23.72%) | 0.653/ 0.558/ 0.881/ **0.045**/ **0.016** | 0.793/ 0.248/ **0.012**/ -/ 0.557 |
| **CVI p5** | 33.575 ± 0.714 | 25.438 ± 1.533 | 26.769 ± 1.121 | 24.667 ± 1.835 | 25.571 ± 1.790 | 28.200 ± 1.082 | **<0.001** (24.24%)**/ <0.001** (20.27%)**/ 0.001** (26.53%)**/ 0.001** (23.84%)**/ 0.011** (16.01%) | 0.610/ 0.483/ 0.079/ **0.026**/ 0.348 | 0.706/ 0.654/ **0.045**/ -/ 0.439 |
| **CVI p6** | 30.900 ± 0.697 | 25.219 ± 1.582 | 21.808 ±1.553 | 21.750 ± 1.951 | 24.524 ± 1.732 | 22.880 ± 1.353 | **0.035** (18.39%)**/ <0.001** (29.42%)**/ 0.001** (29.61%)**/ 0.035** (20.63%)**/ <0.001** (25.95%) | **0.013**/ 0.424/ 0.526/ 0.075/ 0.081 | 0.673/ 0.272/ 0.600/ -/ 0.533 |
| **CVI p7** | 28.825 ± 0.906 | 25.938 ± 1.885 | 21.539 ±1.362 | 22.833 ± 2.679 | 25.571 ± 1.511 | 22.640 ± 2.139 | 1.000 (10.02%)/ **0.004** (25.28%)/ 0.458 (20.79%)/ 1.000 (11.29%)/ 0.092 (21.46%) | 0.361/ 0.993/ 0.906/ **0.044**/ 0.175 | 0.982/ 0.509/ 0.432/ -/ 0.246 |
| **CVI p8** | 29.725 ± 0.920 | 27.469 ±1.829 | 21.385 ±1.632 | 19.583 ± 1.505 | 23.810 ± 1.948 | 21.000 ± 1.694 | 1.000 (7.59%)/ **0.004** (28.06%)/ **0.002** (34.12%)/ 0.166 (19.90%)/ **0.001** (29.35%) | 0.496/ 0.581/ 0.229/ **0.006**/ 0.188 | 0.747/ 0.646/ 0.267/ -/ 0.870 |
| **CVI p9** | 29.050 ± 0.857 | 24.313 ± 1.766 | 21.423 ± 1.653 | 19.667 ± 2.137 | 23.809 ± 1.755 | 21.560 ± 1.449 | 0.199 (16.31%)/ **0.006** (26.25%)**/ 0.007** (32.30%)/ 0.206 (18.04%)/ **0.002** (25.78%) | 0.787/ 0.782/ 0.908/ 0.113/ 0.456 | 0.568/ 0.369/ 0.324/ -/ 0.209 |

Supplementary table 4. Liner correlation analysis of PDs in different groups with BCVA/VF

|  |  | **Layer** | **Part** | **Pearson r** | **p value** |
| --- | --- | --- | --- | --- | --- |
| ***CYP4V2*** | **BCVA** | **SCP** | p1/p2/p3/p4/p5/  p6/p7/p8/p9 | 0.663/0.398/0.265/0.711/0.399/  0.360/0.640/0.763/0.690 | 0.110/0.082/0.258/0.100//0.082/  0.119/0.200/0.850/0.616 |
|  |  | **DCP** | p1/p2/p3/p4/p5/p6/p7/p8/p9 | 0.103/0.272/0.402/0.203/0.277/  0.017/0.387/0.530/0.529 | 0.667/0.246/0.079/0.392/0.237/  0.944/0.092/0.160/0.160 |
|  |  | **CC** | p1/p2/p3/p4/p5/p6/p7/p8/p9 | 0.099/0.096/0.213/0.255/0.057/  0.015/0.368/0.029/0.100 | 0.677/0.687/0.367/0.278/0.811/  0.949/0.110/0.902/0.676 |
|  |  | **MLC** | P1/p3/p7/p8/p9 | 0.202/0.083/0.477/0.31/0.295 | 0.393/0.729/.0.361/0.184/0.207 |
|  | **VF** | **SCP** | p1/p2/p3/p4/p5/p6/p7/p8/p9 | 0.647/0.399/0.090/0.455/0.478/  0.134/0.163/0.563/0.578 | 0.4030/0.253/0.804/0.186/0.162/  0.712/0.652/0.090/0.080 |
|  |  | **DCP** | p1/p2/p3/p4/p5/p6/p7/p8/p9 | 0.162/0.282/0.311/0.261/0.383/  0.056/0.156/0.649/0.692 | 0.655/0.430/0.381/0.466/0.275/  0.879/0.666/0.042/0.027 |
|  |  | **CC** | p1/p2/p3/p4/p5/p6/p7/p8/p9 | 0.082/0.490/0.227/0.258/0.107/  0.095/0.286/0.180/0.257 | 0.822/0.151/0.529/0.471/0.768/  0.793/0.422/0.619/0.473 |
|  |  | **MLC** | P1/ p4/ p5/ p6 | 0.342/0.441/0.391/0.448 | 0.334/0.202/0.264/0.194 |
| ***EYS*** | **BCVA** | **SCP** | P4/p5/p6/P8/P9 | 0.115/0.099/0.051/0.444/0.306 | 0.592/0.646/0.814/0.066/0.145 |
|  |  | **DCP** | p1/p2/p3/p4/p5/p6/p7/p8/p9 | 0.064/0.005/0.114/0.034/0.185/  0.078/0.013/0.286/0.137 | 0.767/0.983/0.596/0.876/0.386/  0.718/0.952/0.176/0.524 |
|  |  | **CC** | p1/p2/p3/p4/p5/p6/p7/p8/p9 | 0.366/0.340/0.404/0.253/0.126/  0.063/0.380/0.493/0.241 | 0.079/0.104/0.050/0.232/0.557/  0.770/0.067/0.014/0.256 |
|  |  | **MLC** | P2/p4/p5/P6/P8/P9 | 0.158/0.288/0.008/0.390/0.023/  0.258 | 0.461/0.172/0.969/0.060/0.915/  0.223 |
|  | **VF** | **SCP** | p1/p2/p3/p4/p5/p6/p7/p8/p9 | 0.403/0.595/0.183/0.289/0.012/  0.164/0.253/0.546/0.245 | 0.219/0.053/0.590/0.389/0.973/  0.630/0.452/0.082/0.469 |
|  |  | **DCP** | p1/p2/p3/p4/p5/p6/p7/p8/p9 | 0.572/0.575/0.690/0.324/0.514/  0.409/0.735/0.722/0.876 | 0.066/0.064/0.190/0.331/0.106/  0.212/0.100/0.120/0.746 |
|  |  | **CC** | p2/p3/p4/p5/p6/p7/p8/p9 | 0.354/0.284/0.029/0.429/0.037/  0.271/0.329/0.370 | 0.286/0.398/0.933/0.188/0.913/  0.420/0.322/0.263 |
|  |  | **MLC** | p1/p2/p3/p5/p7/p8/p9 | 0.176/0.362/0.385/0.143/0.593/  0.484/0.159 | 0.605/0.274/0.242/0.676/0.055/  0.132/0.641 |
| ***PRPH2*** | **BCVA** | **SCP** | p1/p2/p3/p4/p5/p6/p7/p8/p9 | 0.148/0.039/0.073/0.286/0.436/  0.103/0.138/0.400/0.185 | 0.663/0.909/0.831/0.395/0.180/  0.764/0.686/0.223/0.586 |
|  |  | **DCP** | p1/p2/p3/p4/p5/p6/p7/p8/p9 | 0.330/0.334/0.128/0.442/0.329/  0.199/0.460/0.461/0.199 | 0.321/0.315/0.708/0.173/0.324/  0.557/0.155/0.154/0.558 |
|  |  | **CC** | p1/p2/p3/p4/p5/p6/p8/p9 | 0.540/0.250/0.094/0.284/0.218/  0.251/0.315/0.147 | 0.086/0.458/0.782/0.398/0.519/  0.456/0.345/0.667 |
|  |  | **MLC** | p1/p2/p3/p4/p5/p6/p7/p8/p9 | 0.535/0.088/0.293/0.029/0.309/  0.217/0.015/0.191/0.361 | 0.090/0.797/0.381/0.933/0.355/  0.522/0.964/0.574/0.275 |
|  | **VF** | **SCP** | p1/p3/p4/p6/p8/p9 | 0.024/0.262/0.257/0.054/0.580/  0.404 | 0.959/0.570/0.578/0.908/0.173/  0.368 |
|  |  | **DCP** | p1/p2/p3/p4/p5/p6/p7/p8/p9 | 0.081/0.170/0.174/0.311/0.144/  0.028/0.864/0.704/0.098 | 0.863/0.716/0.710/0.498/0.757/  0.952/0.120/0.078/0.834 |
|  |  | **CC** | p1/p2/p3/p4/p5/p6/p7/p8/p9 | 0.236/0.382/0.582/0.027/0.435/  0.295/0.840/0.050/0.094 | 0.610/0.398/0.170/0.954/0.330/  0.521/0.180/0.916/0.840 |
|  |  | **MLC** | p1/p2/p3/p4/p5/p6/p7/p8/p9 | 0.596/0.081/0.329/0.125/0.679/  0.443/0.303/0.583/0.108 | 0.158/0.863/0.471/0.790/0.093/  0.320/0.510/0.170/0.817 |
| ***RPGR*** | **BCVA** | **SCP** | p2/p3/p4/p5/p6/p7/p8/p9 | 0.370/0.272/0.330/0.334/0.234/  0.343/0.343/0.193 | 0.159/0.308/0.212/0.207/0.384/  0.193/0.194/0.475 |
|  |  | **DCP** | p1/p2/p3/p4/p5/p6/p7/p8/p9 | 0.106/0.121/0.163/0.070/0.107/  0.146/0.001/0.031/0.007 | 0.697/0.657/0.546/0.798/0.692/  0.589/0.996/0.908/0.978 |
|  |  | **CC** | p1/p2/p3/p6/p9 | 0.305/0.027/0.305/0.083/0.431 | 0.252/0.921/0.251/0.760/0.095 |
| ***USH2A*** | **BCVA** | **SCP** | p1/p2/p4/p5/p6/p7/p8/p9 | 0.040/0.398/0.181/0.465/0.156/  0.455/0.311/0.119 | 0.863/0.074/0.433/0.663/0.500/  0.380/0.171/0.608 |
|  |  | **DCP** | p1/p2/p3/p4/p5/p6/p7/p8/p9 | 0.073/0.372/0.062/0.117/0.429/  0.063/0.351/0.317/0.120 | 0.752/0.097/0.790/0.612/0.052/  0.787/0.119/0.161/0.605 |
|  |  | **CC** | p1/p2/p3/p4/p5/p6/p7/p8/p9 | 0.510/0.377/0.445/0.325/0.113/  0.151/0.261/0.126/0.468 | 0.180/0.092/0.430/0.151/0.626/  0.514/0.253/0.585/0.330 |
|  |  | **MLC** | p1/p3/p4/p5/p6/p7/p8/p9 | 0.551/0.652/0.837/0.262/0.540/  0.629/0.646/0.356 | 0.150/0.100/0.100/0.252/0.120/  0.200/0.200/0.113 |
|  | **VF** | **SCP** | p1/p2/p3/p4/p5/p6/p7/p8/p9 | 0.120/0.001/0.401/0.027/0.055/  0.243/0.151/0.053/0.128 | 0.637/0.996/0.099/0.916/0.829/  0.332/0.549/0.834/0.612 |
|  |  | **DCP** | p1/p2/p3/p4/p5/p6/p7/p8/p9 | 0.089/0.029/0.368/0.019/0.097/  0.286/0.147/0.092/0.023 | 0.726/0.909/0.133/0.941/0.703/  0.250/0.560/0.716/0.928 |
|  |  | **CC** | p1/p2/p3/p4/p5/p6/p7/p8/p9 | 0.288/0.151/0.308/0.136/0.179/  0.078/0.041/0.218/0.283 | 0.247/0.549/0.213/0.590/0.476/  0.758/0.873/0.384/0.255 |
|  |  | **MLC** | p1/p2/p3/p4/p5/p6/p7/p8/p9 | 0.038/0.082/0.040/0.160/0.135/  0.181/0.287/0.109/0.216 | 0.880/0.748/0.876/0.527/0.594/  0.472/0.248/0.667/0.389 |
